# Quantifying CD4 receptor protein in two human CD4+ lymphocyte preparations for quantitative flow cytometry

**DOI:** 10.1186/1559-0275-11-43

**Published:** 2014-12-11

**Authors:** Meiyao Wang, Martin Misakian, Hua-Jun He, Peter Bajcsy, Fatima Abbasi, Jeffrey M Davis, Kenneth D Cole, Illarion V Turko, Lili Wang

**Affiliations:** Institute for Bioscience and Biotechnology Research, University of Maryland, 9600 Gudelsky Drive, Rockville, MD 20850 USA; Biomolecular Measurement Division, National Institute of Standards and Technology (NIST), Gaithersburg, MD 20899 USA; Quantum Measurements Division, NIST, 100 Bureau Drive, Stop 8312, Gaithersburg, MD 20899 USA; Biosystems and Biomaterials Division, NIST, 100 Bureau Drive, Stop 8312, Gaithersburg, MD 20899 USA; Software and Systems Division, NIST, 100 Bureau Drive, Stop 8312, Gaithersburg, MD 20899 USA; Laboratory of Stem Cell Biology, Cellular and Tissue Therapy Branch, Division of Cell and Gene Therapies, CBER FDA, 8800 Rockville Pike, Bethesda, MD 20892 USA; Materials Measurement Science Division, NIST, 100 Bureau Drive, Stop 8312, Gaithersburg, MD 20899 USA

**Keywords:** Cryopreserved peripheral blood mononuclear cells (PBMC), Lyophilized Cyto-Trol™, CD4 receptor density, Flow cytometry, Multiple reaction monitoring (MRM) mass spectrometry (MS), Scanning electron microscopy (SEM), Microvilli, Cell surface area

## Abstract

**Background:**

In our previous study that characterized different human CD4+ lymphocyte preparations, it was found that both commercially available cryopreserved peripheral blood mononuclear cells (PBMC) and a commercially available lyophilized PBMC (Cyto-Trol™) preparation fulfilled a set of criteria for serving as biological calibrators for quantitative flow cytometry. However, the biomarker CD4 protein expression level measured for T helper cells from Cyto-Trol was about 16% lower than those for cryopreserved PBMC and fresh whole blood using flow cytometry and mass cytometry. A primary reason was hypothesized to be due to steric interference in anti- CD4 antibody binding to the smaller sized lyophilized control cells.

**Method:**

Targeted multiple reaction monitoring (MRM) mass spectrometry (MS) is used to quantify the copy number of CD4 receptor protein per CD4+ lymphocyte. Scanning electron microscopy (SEM) is utilized to assist searching the underlying reasons for the observed difference in CD4 receptor copy number per cell determined by MRM MS and CD4 expression measured previously by flow cytometry.

**Results:**

The copy number of CD4 receptor proteins on the surface of the CD4+ lymphocyte in cryopreserved PBMCs and in lyophilized control cells is determined to be (1.45 ± 0.09) × 10^5^ and (0.85 ± 0.11) × 10^5^, respectively, averaged over four signature peptides using MRM MS. In comparison with cryopreserved PBMCs, there are more variations in the CD4 copy number in lyophilized control cells determined based on each signature peptide. SEM images of CD4+ lymphocytes from lyophilized control cells are very different when compared to the CD4+ T cells from whole blood and cryopreserved PBMC.

**Conclusion:**

Because of the lyophilization process applied to Cyto-Trol control cells, a lower CD4 density value, defined as the copy number of CD4 receptors per CD4+ lymphocyte, averaged over three different production lots is most likely explained by the loss of the CD4 receptors on damaged and/or broken microvilli where CD4 receptors reside. Steric hindrance of antibody binding and the association of CD4 receptors with other biomolecules likely contribute significantly to the nearly 50% lower CD4 receptor density value for cryopreserved PBMC determined from flow cytometry compared to the value obtained from MRM MS.

**Electronic supplementary material:**

The online version of this article (doi:10.1186/1559-0275-11-43) contains supplementary material, which is available to authorized users.

## Background

Cluster of differentiation 4 (CD4) is a glycoprotein expressed on the surface of many different types of immune cells, e.g., T helper cells and monocytes. As a coreceptor, CD4 assists the T cell receptor to initiate T cell activation and counter attack foreign peptides processed by antigen presenting cells. In human T lymphocytes, the CD4 receptor protein encoded by the CD4 gene consists of four distinct extracellular domains (D1 to D4), a transmembrane domain and a short intracellular tail [1]. Defining T helper cells in immunophenotyping is carried out by using anti-human CD4 monoclonal antibodies against part of the four extracellular domains of the receptor. Numerous reports in the literature indicate that the CD4 expression level on normal human T helper cells is fairly consistent [2–5]. Therefore the CD4 receptor protein can serve as a biological calibrator for quantification of the surface and intracellular proteins of human immune cells using flow cytometry. Biological calibrators are essential for the transformation of a linear fluorescence intensity scale obtained with fluorescent calibration microspheres to an antibody bound per cell (ABC) scale [6, 7].

In our previous study that characterized different human CD4+ lymphocyte preparations [8], it was found that both cryopreserved peripheral blood mononuclear cells (PBMC) and lyophilized control cells (Cyto-Trol™) generally fulfilled a set of criteria for biological calibrators. The criteria include reproducibility and tightness of detected CD4 expression, close physical and chemical resemblance of fresh blood samples, and long term sample stability under widely used and common storage conditions. The 16% lower CD4 expression level measured for the lyophilized control cells compared to cryopreserved PBMC and fresh whole blood using both quantitative flow cytometry and mass cytometry, was previously hypothesized to be due to steric interference associated with anti- CD4 antibody binding to the smaller size lyophilized control cells. That is, the average diameter of the CD4+ lymophocytes is 6.3 ± 0.4 μm for the lyophilized control cells (Cyto-Trol™) and 7.5 ± 0.4 μm for cryopreserved PBMCs, respectively. In the present study, both targeted mass spectrometry by liquid chromatography (LC) MRM and scanning electron microscopy (SEM) are used to determine the underlying reasons for the observed difference in CD4 expression.

## Results

### Quantification of endogenous CD4 receptors by MRM MS

A new batch of the isotope labeled internal standard CD4 protein for targeted mass spectrometry-based quantification was commercially obtained from Origene. This internal standard CD4 has the full-length endogenous human CD4 sequence given in the Additional file 1, a recombinant peptide on each end of the endogenous CD4 protein, and is isotope-labeled with ^15^N and ^13^C on each lysine and arginine residue. A detailed characterization of the MS standard protein was performed using our previous published method [9]. The concentration of the stable isotope labeled standard CD4, *N*_*iso*_, was calculated according to Eq. 1 given below,

1

*I*_*iso*_ and *I*_*r*_ refer to the intensity of the isotope labeled peptide peak and intensity of a recombinant CD4 protein (rCD4) (obtained from NIH AIDS Research & Reference Reagent Program with a known concentration obtained from amino acid analysis), respectively. *I*_*n-iso*_ corresponds to the intensity of the total non-isotope labeled peptide peak detected and the constant, 0.31 is the ratio of the non-labeled to the labeled peptide obtained from the internal standard CD4. *N*_*r*_ is the mol/L concentration of rCD4 derived from the amino acid analysis. A final concentration of 0.16 pmol/μL and isotope incorporation of 76.2% was applied for the present endogenous CD4 quantification. The endogenous CD4 protein concentration, *N*_*end*_ was derived in the same fashion from the ratio of the non-labeled and labeled MRM transition peak intensities multiplied by the known amount of standard spiked into the sample on the basis of Eq. 2,
2

*I*_*end*_ stands for the intensity of the endogenous CD4 peptide peak.

Target peptide selection for MS quantification was based on several factors, i.e., ion stability, favorable transition intensities, and minimum matrix effects. These factors were individually tested empirically. To avoid the bias of any single peptide, the CD4 MRM quantification in any given sample was based on the average value of a total of 4 signature peptides (P1: ILGNQGSFLTK; P2: SLWDQGNFPLIIK; P3: ASSIVYK; P4: ATQLQK, defined in Additional file 1). Each peptide was monitored by 3 pairs of the precursor peptide ion and specific fragment ion (a so called “transition”) [9]. The mean value of 4 peptides (P1 to P4) was taken as the CD4 density in each measured sample. Considering the sample to sample variation due to cell preparation, sample processing and analysis, we performed multiple biological sample replications for quantitative analysis of the CD4+ T cells from each cell source (5 replicates for lyophilized Cyto-Trol cells and 3 replicates for cryopreserved PBMC). Because no outlier was found by Grubbs test, the mean value of these sample replicates was taken as the CD4 receptor protein density. The results of the endogenous CD4 quantification are summarized in Table 1. The copy number of CD4 receptor proteins on the surface of the CD4+ lymphocyte in cryopreserved PBMC and in lyophilized control cells is (1.45 ± 0.09) × 10^5^ and (0.85 ± 0.11) × 10^5^, respectively. The CD4 receptor density from the lyophilized control cells is significantly lower than the value from cryopreserved PBMC.

**Table 1:** **CD4 density per CD4+ lymphocyte and associated one standard deviation of the mean obtained for Cyto-Trol from three different production lots and for cryopreserved PBMC from five different production lots**

	Cyto-Trol						PBMC			
Replicate	1	2	3	4	5	Mean Density (×10 ^5^)	1	2	3	Mean Density (×10 ^5^)
Cell # (×10^6^)	5.25	6.00	6.00	6.00	6.00	-	4.39	4.26	4.32	-
Density averaged over P1 to P4 (×10^5^)	0.85	0.89	0.97	0.67	0.86	0.85(0.11)	1.48	1.52	1.34	1.45(0.09)

For each sample replicate, the CD4+ cell number was provided and the CD4 density value was averaged over 4 peptides, P1 to P4 (See Additional file 1).

**Figure 1:**
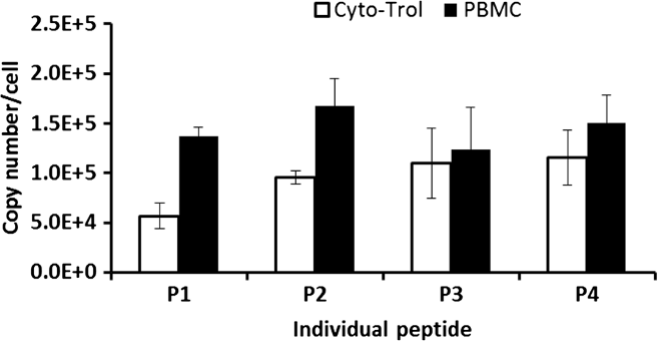
**CD4 density and associated one standard deviation of the mean obtained on the basis of different peptides, P1 to P4 by MRM MS for CD4+ T cells from Cyto-Trol and cryopreserved PBMC.**

We further analyzed the CD4 copy number obtained from each peptide in cryopreserved PBMC and in Cyto-Trol (Figure 1). From 5 Cyto-Trol replicates, the peptide P1, which is located close to the protein N-terminal of the CD4 protein and furthest away from cell membrane, gives a lower density value [(0.57 ± 0.12) × 10^5^] than the other three peptides [P2: (0.95 ± 0.07) × 10^5^; P3: (1.10 ± 0.33) × 10^5^; P4: (1.16 ± 0.28) × 10^5^]. However, this low value is not identified as an outlier by Grubbs test on only four density values determined from four peptides. By comparison, the density value obtained from the same P1 peptide from 3 PBMC replicates shows no apparent difference from the values obtained by the other three peptides. Interestingly, for Cyto-Trol replicates, the density value increases monotonically from P1 to P4, unlike the value for PBMC replicates showing no specific trend.

### Scanning electron microscopy measurements

SEM images of the CD4+ T cells purified from cryopreserved PBMC, normal donor whole blood and lyophilized Cyto-Trol cells were obtained using sample preparation procedures described in the ‘Materials and Methods’. There was no significant difference observed for the same sample types when two different procedures were applied at the last stage of the cover glasses coated with CD4+ lymphocytes, critical point drying with liquid carbon dioxide or immersing in hexamethyldisilazane (HMDS). The CD4+ T cell images are described below in terms of their overall appearance and approximate diameters defined as the average of two orthogonal measurements.

**Figure 2:**
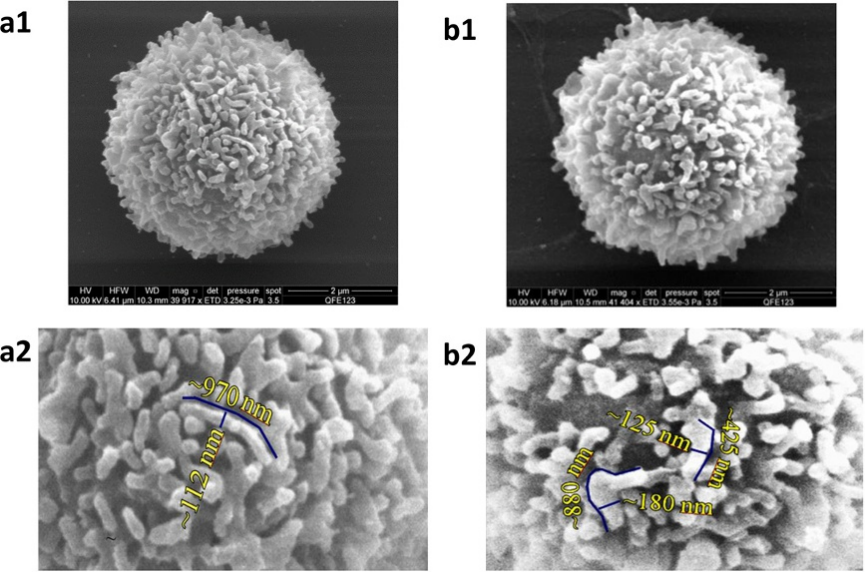
**Representative electron microscope images of CD4+ T cells purified from fresh normal donor whole blood (a1, a total of 14 images were recorded) and cryopreserved PBMC (b1, 7 images were recorded) along with the respective magnified central portions of the CD4+ T cell surfaces (a2 for whole blood and b2 for cryopreserved PBMC, respectively), showing approximate diameters and lengths of some microvilli.**

A representative image of the CD4+ lymphocytes from whole blood is shown in Figure 2(a1) and showed no significant differences from cryopreserved PBMC shown in Figure 2(b1). Fourteen images acquired for CD4+ lymphocytes from whole blood and 7 images for cryopreserved PBMC indicate that the average diameter of the CD4+ cells is about 4.7 μm for whole blood and 4.6 μm for cryopreserved PBMC, respectively, and their microvilli are densely packed. Figure 2(a2) and (b2) display magnified central portions of the CD4+ cell surface, providing a clearer view of the microvilli structure on both types of cell surface. The microvilli exhibit diameters of 100 nm to 200 nm and lengths of 500 nm to 1000 nm estimated manually from the sample images. It is expected that closely spaced microvilli can hinder the antibody binding or quench the emission of fluorescent molecules.

**Figure 3:**
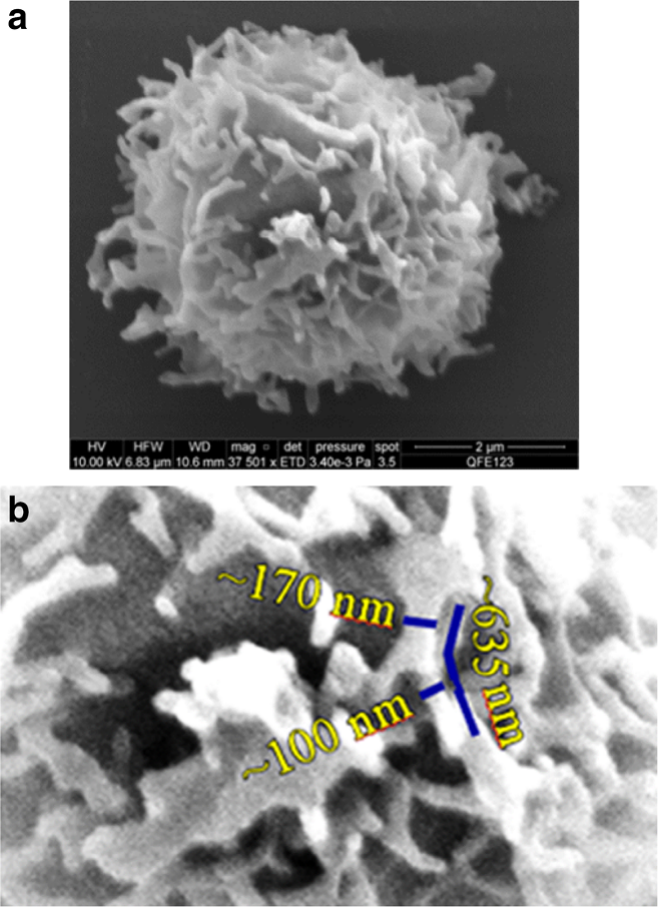
**(a) A representative scanning electron microscope image of CD4+ T cells purified from Cyto-Trol control cells (a total of 15 images were recorded): Elongated microvilli can be seen near the perimeter of the cell; (b) A magnified central portion of the CD4+ cell surface showing approximate diameters and length of microvilli.**

**Figure 4:**
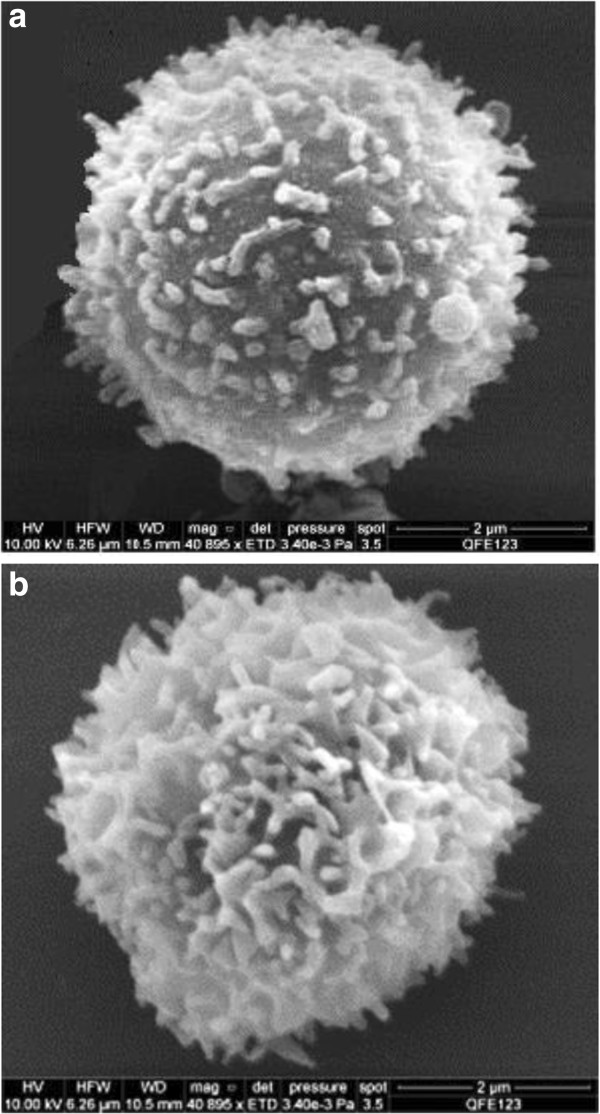
**A few exceptional images other than that shown in Figure** 3 **a were observed for CD4+ T cells from lyophilized Cyto-Trol: (a) the entangled microvilli were replaced with truncated and sparsely populated microvilli in two instances; (b) densely populated microvilli showed early signs of entanglement.**

An example of a CD4+ lymphocyte image from lyophilized Cyto-Trol cells is shown in Figure 3a and is very different when compared to the CD4+ cells from whole blood and PBMCs shown in Figure 2. The lyophilizing process is evidently responsible for their generally altered and more tangled appearance. Interestingly, the microvilli can be elongated (~2.5 μm, Figure 3a, top center), or as shown in Figure 3b, the microvilli can have similar diameters except near their narrowed tips. The average diameter of the CD4+ cells from Cyto-Trol is about 4.8 μm, similar to those for whole blood and PBMCs (4.6-4.7 μm). The similar sizes of the CD4+ T cells obtained from three different sample sources are mostly due to the cell processing procedure applied, e.g., dehydration and critical point drying. Of the total of 15 images obtained there were few exceptions to the thin and entangled appearance of the lyophilized CD4+ T cells shown in Figure 3. In two cases the entangled microvilli were replaced with fewer truncated and sparsely populated microvilli (e.g., Figure 4a). The remaining exception in one case is shown in Figure 4b. That is, the somewhat densely populated microvilli showed early signs of entanglement (Figure 4b).

### Quantitative comparison of SEM image characteristics

**Figure 5:**
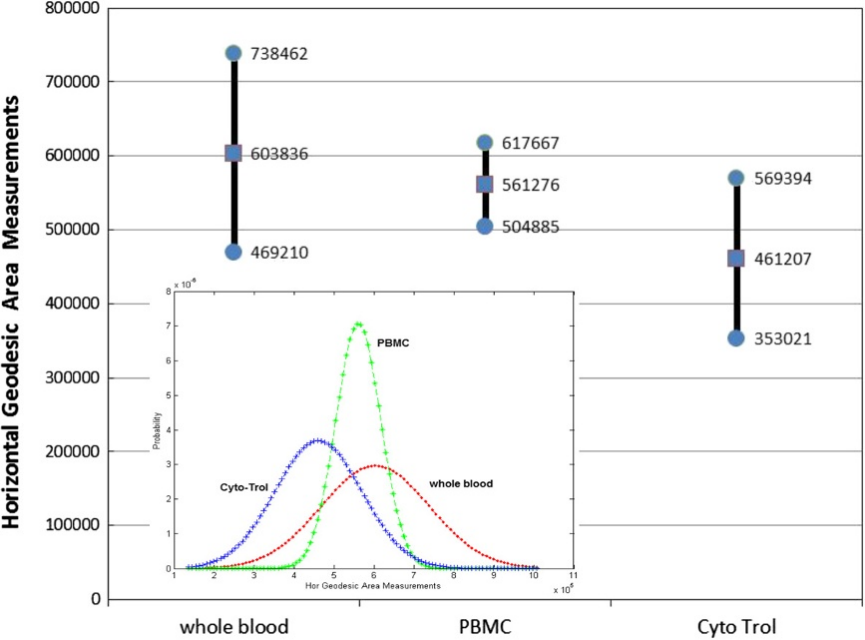
**Characterization of horizontal geodesic surface area in pixel count of the three types of cells by Gaussian probability distribution function (PDF): the sample mean and standard deviation values per cell type are plotted in the main graph; the relative positions of the three PDFs are displayed in the inset.**

In order to quantify visually observed differences in SEM images, we have analyzed 12 images of whole blood (2 images with size ruler shown in the image files were omitted from the analyses), 7 images of cryopreserved PBMC, and 15 images of Cyto-Trol. To avoid cell segmentation challenges, we extracted automatically 600 × 600 pixels from the center of each image of size 2048 × 1887 since all SEM images are centered on a cell and a cell radius is about 680 pixels. In order to minimize the influence of image acquisition noise on surface area measurements defined in Eq. 3 in the ‘Materials and Methods’ on ‘Comparison of SEM Image Characteristics’, we applied a morphological filter with a square kernel of size 7 pixels to all images. After computing a geodesic surface area in pixel count per extracted image subarea (each pixel corresponds to an area of specific μm^2^/pixel), we computed sample mean and standard deviation of the surface area measurements per image label (whole blood, PBMC and Cyto-Trol). Figure 5 shows the resulting statistics and their corresponding Gaussian probability distribution functions (PDFs). The mean surface area for CD4+ T cells from Cyto-Trol (461207) is smaller than the surface area of CD4+ T cells from cryopreserved PBMC (561276) and the area of CD4+ lymphocytes from whole blood (603836), suggesting fewer and/or shorter microvillis on the surface of CD4+ T cells from Cyto-Trol as observed visually. We plotted only statistics of surface area measured along the horizontal direction since the correlation between vertical and horizontal values is 0.98.

## Discussion

The CD4 receptor density on T helper cells from cryopreserved PBMC is determined to be (1.45 ± 0.09) × 10^5^ copies/cell using the MRM MS method (Table 1). This result is nearly identical to the value [(1.46 ± 0.03) × 10^5^ copies/cell] reported earlier for the commercial cryopreserved human CD4+ T cells with 98.5% purity [9]. Due to the lower number of CD4+ cells enriched from cryopreserved PBMC in the present study, the standard deviation of the MRM measurements is larger than that reported previously though the same fragment peptides were detected and used for CD4 density value determinations. Nonetheless, the consistent CD4 receptor density values of two different sample preparations demonstrate the robustness of the MRM MS method developed in-house.

It is surprising that the CD4 receptor density value for CD4+ T cells from PBMC (1.45 × 10^5^ copies/cell) is about 50% greater than the value determined from flow cytometry measurements (9.8 × 10^4^ copies/cell) where unimolar anti-CD4 PE antibodies were used to determine the ABC value on a human T helper cells [8]. It is noted that the CD4 density value determined from flow cytometry measurements is calculated as twice of the ABC value because each anti-CD4 monoclonal antibody (clone Leu-3a) binds two CD4 receptors through the extracellular domain, D1 of CD4 receptor protein on human T lymphocytes [5]. The SEM images of CD4+ lymphocytes from both cryopreserved PBMC and fresh whole blood show dense microvilli structures on cell surfaces. As a result, it is likely that some steric hindrance of antibody binding exists and the association of CD4 receptors with other biomolecules [10–12], i.e., CCR5, CXCR4 and lipid rafts, may further prevent the anti-CD4 antibody binding.

Compared to the CD4 receptor density on CD4+ T cells from cryopreserved PBMC, the density value obtained for lyophilized Cyto-Trol cells is lower (0.57 × 10^5^ copies/cell from P1, 0.95 × 10^5^ copies/cell from P2, 1.10 × 10^5^ copies/cell from P3, 1.16 × 10^5^ copies/cell from P4). Because peptides P1 and P2 reside in the CD4 extracellular domain D1 (See Additional file 1) where anti-CD4 antibodies (clone Leu-3a) are bound in flow cytometry measurements, it is logical to compare the CD4 density values obtained from P1 and P2 to the value estimated from flow cytometry. The values from P1 and P2 are reasonably consistent with that estimated from flow cytometry measurements (8.2 × 10^4^ copies/cell) [8]. It is understood that not all CD4 receptors on T cells are accessible for anti-CD4 antibody binding. The general agreement of the two results from two different measurement techniques implies that steric hindrance and denaturation stresses of the lyophilization process on the paratope of CD4 receptor proteins [13] are marginal for preventing the antibody binding to the CD4 receptors. The SEM images of CD4+ T cells from Cyto-Trol, with some exceptions, show elongated and entangled microvilli structures unlike those observed from cryopreserved and freshly prepared PBMC. Based on the observation that the CD4 density values for Cyto-Trol are much lower than the value for PBMC (1.45 × 10^5^ copies/cell), and Cyto-Trol was produced from normal donor PBMC just like cryopreserved PBMC, one could postulate that CD4 receptors on microvilli might be damaged during the lyophilization process (Figure 3a). In the quantification of multiple peptides using the MRM MS method, we observed that the quantity of the peptide P1 (ILGNQGSFLTK) that is close to the N terminal of the CD4 receptor protein was consistently lower than the other three peptides in the middle of the receptor protein (Figure 1). This observation does support the hypothesis that damage of the CD4 receptor proteins may have occurred in the lyophilzation process. The reported possible glycosylation on the asparagine near the peptide P4 (ATQLQK*N*) may generate peptide missed cleavage by trypsin, resulting in an underestimation of P4 level [14]. This will only increase the difference between P1 and P4 and support the same conclusion. On the basis of Spellman et al’s report [14] and our previous quantification result [9], the potential glycosylation effect is negligible. The lower CD4 density value from Cyto-Trol cells measured by both flow cytometry and MRM MS methods is most likely explained by the damage of the CD4 receptor proteins or broken microvilli (Figures 3a and 4a).

Based on the quantitative surface area measurements from SEM images (Figure 5), we evaluated a hypothesis that the surface area measurements of Cyto-Trol and PBMC came from the same probability distribution (PDF) as the measurements of whole blood. We modeled the surface area measurements per cell class with normal PDF that is uniquely described by its mean and variance (inset of Figure 5). One can then evaluate hypotheses using a two-tailed t-test of mean, a chi-squared test of variance or Kolmogorov-Smirnov test of PDF shape. For example, for the two-tailed t-test with a significance level equal to 0.05, we cannot reject the null hypothesis that the sample mean of PBMC is equal to the mean of whole blood (p-value is 0.0928 > 0.05). In contrast, we can reject the null hypothesis that the sample mean of Cyto-Trol is equal to the mean of whole blood (p-value is 0.0002 < 0.05). Intuitively, if data points from a class (Cyto-Trol or PBMC) are coming statistically from the whole blood then the PDFs of Cyto-Trol and PBMC would have very close values of sample means and variances, and hence a significant overlap with the whole blood PDF (ideally the PDFs have the overlap equal to one). Thus, the fact that the overlap of the Cyto-Trol PDF with the whole blood PDF (0.3347) is smaller than the overlap of the PBMC PDF with the whole blood PDF (0.8621) implies that PBMC data points are more likely coming from the whole blood PDF than the Cyto-Trol data points. Although the number of images per label is relative low, one could imply that the Cyto-Trol production protocol causes the cells to change their surface roughness 2.6 times more likely than the PBMC protocol in comparison to the whole blood label. These types of quantitative image analyses could lead to establishing better understanding for morphological deviation of any cell production protocol applied to whole blood and assisting the production of cell reference materials with known biomarker expression levels for quantitative immunophenotyping.

## Conclusions

In this study, the MRM MS and SEM measurements are used to assess two human blood cell preparations in search of optimal cell reference materials for quantitative flow cytometry that are more stable and easier to maintain than fresh whole blood. Because of the lyophilization process, the CD4 density value on CD4+ lymphocytes from Cyto-Trol cells is lower than the value from cryopreserved PBMC, most likely explained by the truncation of the CD4 receptor proteins and damaged and/or broken microvilli where CD4 receptors reside. On the other hand, steric hindrance of antibody binding and the association of CD4 receptors with other biomolecules likely contribute significantly to the close to 50% lower CD4 receptor density value for cryopreserved PBMC determined from flow cytometry than the value obtained from MRM MS.

The consistent CD4 expression on T cells in normal donor PBMC, serving as the biological control enhances the reliability of clinical diagnostics and immunotherapies. This CD4 receptor protein also plays a crucial role in the progression of HIV-1 viral infection in that the gp120 viral protein binds to the CD4 receptor on T cells, leading to the viral entry and cell disintegration [15]. Though numerous efforts have been put in the development of vaccines against the infection, there is limited success largely because of the complexity of the viral infection process and limited robust measurement techniques supporting the understanding of the underlying mechanisms of trial vaccines. The three powerful techniques used in this study, flow cytometry, MRM MS and scanning electron microscopy, allowed us to better understand the changes caused by the lyophilization process on CD4+ lymphocytes. These techniques would enable the measurements of CD4 receptor density and the number of anti-CD4 monoclonal antibodies, e.g., Ibalizumab [16] and Bispecific Ibalizumab [17], bound to cells bearing CD4 receptors. These measurements would greatly help to shed light on the underlying mechanisms of two trial vaccines for the treatment and prevention of HIV-1. Furthermore, down modulated CD4 cell surface expression and subcellular localization [18], and depletion of the surface CD4 protein [19] have been reported in the literature in some cases of HIV infection. It would certainly be more challenging to apply these techniques to measure internalized CD4 proteins in different cell compartments.

## Materials and methods

### Reagents

Anonymous heparinized normal donor samples were obtained from NIH’s Department of Transfusion Medicine and was exempted for research use by its institutional review boards (IRB). Cyto-Trol kits including Cyto-Trol™ control cells and reconstitution buffer from three different production lots were obtained from Beckman Coulter (Fullerton, CA). Cryopreserved PBMC (Catalog Number: CTL-UP1, five different production lots) and anti-aggregate wash supplement 20× (Catalog Number: CTL-AA-001) were purchased from Cellular Technology Ltd. (Shaker Heights, OH). Anti-CD4 FITC monoclonal antibody and anti-CD3 APC were purchased from BD Biosciences (San Jose, CA). Anti-CD14 Pacific Blue (PB) monoclonal antibody and Dynabeads® Untouched™ Human CD4 T Cells Kit were purchased from Invitrogen (Carlsbad, CA). An isotope labeled CD4 mass spectrometry standard protein was obtained from OriGene Technologies (Rockville, MD). Isotope labels (^13^C and ^15^ N) in the CD4 standard protein were introduced on arginine and lysine residues. All chemicals and reagents, unless indicated specifically, were from Sigma-Aldrich Inc.

### Preparation of human CD4+ T cells

The cryopreserved PBMC were thawed following a protocol provided by Cellular Technology that was described in detail in the previous study [8]. The lyophilized control cells in a vial were reconstituted in 1 mL of reconstitution buffer provided in the Cyto-Trol kit. Both thawed cryopreserved PBMCs and reconstituted lyophilized control cells were washed once and resuspended in phosphate-buffered saline (PBS) with 1% fetal bovine serum (FBS). The cell number was counted by using a hemocytometer after trypan blue staining.

For the MRM measurements, human CD4+ lymphocytes from cryopreserved PBMC and lyophilized control cells were obtained through a negative selection by using Dynabeads Untouched Human CD4 T Cells with a modified enrichment protocol. Briefly, 100 μL heat inactivated FBS was added to 4 × 10^7^ PBMCs in 300 μL isolation buffer [PBS supplemented with 0.1% bovine serum albumin (BSA) and 2 mM EDTA] in a 2 mL centrifuge tube and was followed by addition of 100 μL antibody mix. The antibody mix contains mouse IgG antibodies towards human CD8, CD14, CD16 (specific for CD16a and CD16b), CD19, CD36, CD56, CDw123 and CD235a. The mixture in the tube was placed on an orbital shaker at 30-60 rpm and incubated for 1 hr at 4°C. After the mixture was washed twice with 1.2 mL isolation buffer, the cells were resuspended in 300 μL isolation buffer; 500 μL pre-washed Dynabeads were added followed by incubation for 30 min on a gentle rotating shaker at room temperature. The mixture was transferred to a 15 mL centrifuge tube with isolation buffer to reach a final volume of 3 mL. The bead-bound cells were mixed thoroughly with a pipette that had a narrow tip opening to avoid foaming. The tube was placed in a 15 mL/50 mL tube magnet from Qiagen (Valencia, CA) for 2 min and the supernatant containing the human CD4+ T cells were transferred to a new 15 mL tube. An additional 4 mL isolation buffer was added to the tube containing Dynabeads and the bead-bound cells were resuspended thoroughly with a pipette as described above. This tube was placed in the magnet for 2 min, and the supernatant was collected and combined with the previous supernatant. Lastly, the combined supernatant tube was placed in the magnet for 2 min to further remove residual magnet beads. The supernatant was centrifuged at 400 g for 10 min, and the supernatant was discarded. The purified cells were washed once and resuspended in PBS with 1% FBS. The total number of the purified cells including CD4- and CD4+ T cells and residual monocytes was counted at least three times using the hemocytometer and recorded.

The purified cells (1 × 10^5^) were stained with 25 μL anti-CD4 FITC, 5 μL CD3 APC and 6 μL anti-CD14 PB to account for contribution of CD4 receptor proteins from the residual monocytes in the purified cells. After one wash with 1% FBS in PBS, the stained cells were resuspended in 0.5 mL PBS with 1% FBS and run on a FACSAria II flow sorter. For the purified cells from cryopreserved PBMC, the percentage of CD4+ cells was 93.9 ± 0.9% from three replicates and the percentage of the monocytes with low CD4 expression in the CD4+ cells was 1.2 ± 0.1% from three replicates. For the MRM measurements, the number of CD4+ cells was taken as the product of the total cell number obtained by hemocytometer and 0.939. On the basis of the fluorescence intensity that is assumed to be proportional to the CD4 receptor density and number of the residual monocytes with low CD4 expression and normal CD4+ T cells, a correction factor (1.01) was applied to the CD4 receptor density value derived from the MS measurements for all CD4+ cells to obtain the CD4 density value for just CD4+ T cells.

For the purified cells from the lyophilized control cells (Cyto-Trol™), the percentage of CD4+ cells was 68.5 ± 0.5% from three replicates and the percentage of the residual monocytes with low CD4 expression in the CD4+ cells was 7.9 ± 0.4% from three replicates. For the MS measurements, the number of CD4+ cells was taken as the product of the total cell number obtained by hemocytometer and 0.685. Based on the fluorescence intensity and number of the monocytes with low CD4 expression and normal CD4+ T cells, a correction factor (1.06) was applied to the CD4 receptor density derived from the MS measurements for all CD4+ cells to obtain the CD4 density value for only the CD4+ T cells.

### Sample processing for MRM measurements

The preparation procedure of human CD4+ T cells for MRM measurements is described in our previous report [9] with minor revision. Briefly, the isotope labeled full-length internal standard CD4 was mixed with a known number of human T cells in 150 μL of 25 mmol/L ammonium bicarbonate buffer (Abb), pH 7.9, with 2% SDS. The cell and protein mixture was lysed by brief sonication at 20 W using three 10 s continuous cycles (Sonicator 3000 from Misonix, Farmingdale, NY). The cysteine reduction and alkylation of the proteins were carried out by the treatment with 20 mmol/L DTT at room temperature for 60 min followed by incubation with 50 mmol/L iodoacetamide for 60 min. The cell lysate was centrifuged at 175,000 g for 30 min to remove insoluble fragments. Chloroform/methanol treatment [9, 20] was then performed to precipitate proteins from supernatant and to remove salts and lipids. The precipitated protein mixture was reconstituted in 100 μL of 25 mmol/L Abb containing 0.1% RapiGest followed by protease digestion using trypsin [Sequence Grade Modified, Promega, 1:50 (w:w) trypsin: protein] overnight at 37°C. After enzymatic digestion, the sample was treated with 0.5% trifluoroacetic acid for 30 min at 37°C and centrifuged at 175,000 g for 30 min. The supernatant, which contains soluble peptides, was transferred to a new microcentrifuge tube and dried by vacuum centrifugation (Eppendorf AG, Hamburg, Germany) for subsequent mass spectrometry analysis.

### Nano-LC-MRM analysis

The digested peptides were reconstituted in Milli-Q H_2_O with 3% acetonitrile (ACN) containing 0.1% formic acid followed by nano-LC-MRM analysis. Peptide separation and mass spectrometry analysis were performed on a hybrid triple quadrupole/linear ion trap mass spectrometer (4000 QTRAP, ABI/MDS-SCIEX) coupled to an Eksigent nanoLC-2D system (Dublin, CA). Peptides were separated and eluted at a flow rate of 300 nL/min over a 30 min gradient of acetonitrile from 15% to 35% in H_2_O containing 0.1% formic acid using an Eksigent cHiPLC- nanoflex system equipped with a nano cHiPLC column, 15 cm × 75 μm, packed with ReproSil-Pur C18-AQ, 3 μm (Dr. Maisch, Germany). The eluted peptides were directed into the mass spectrometer with a nanospray source. The subsequent MRM detection of CD4 signature peptides was performed in the positive ion mode with the following key parameters: an ion spray voltage of 2,300 V, curtain gas of 18 psi, source gas of 30 psi, interface heating temperature of 170°C, declustering potential of 76 V for +2 precursor ions and 65 V for +3 precursor ions, collision cell exit potential of 16 V for +2 precursor ions and 13 V for +3 precursor ions, and dwell time of 40 ms for each transition pair. The collision energy of each target transition was optimized empirically as reported in the previous study [9]. To develop the MRM method, peptide selection was based on several criteria including peptide mass is preferably between 700-2500 Da, and cysteine/methionine containing peptides are avoided for quantification purpose. Since the detectable ions of different peptides from a single protein can be different in different mass spectrometers, we selected and optimized the target CD4 peptides and working MS parameters based on favorable transition peak intensities, stable retention times, and minimum biological matrix effects. Considering the complexity of cell lysate, the proportional intensity ratios of the multiple target transitions of the selected peptides from standard CD4 and the counterpart in cell lysate confirmed minimal interference from the biological matrix. Each selected peptide was confirmed as a unique CD4 peptide by sequence blast against human non-redundant genome database (NCBI).

The mass spectrometer was operated using Analyst 1.5.1 (AB SCIEN). Calibration curves showed linearity and low scatter over the range of 0.1 – 5 pmol/μL tested for the internal standard. The endogenous CD4 protein concentration was derived from the ratio of the non-labeled and labeled MRM peak intensities multiplied by the known amount of the internal standard. The identities of the selected peptides were confirmed based on the two parameters of the internal standard run under the same conditions, the retention time of the given peptide and the proportional ratio among the MRM transitions. Each pair of transition from a given peptide was treated as an independent measure for the peptide, resulting in a concentration value expressed as copy number of the quantified peptide per cell. Analysis of each selected signature peptide was based on the mean value of multiple transitions from the peptide. Four signature peptides were employed to evaluate the endogenous CD4 concentration. Each sample was measured in triplicate and a total of at least three cell lysate replicates were prepared and measured independently.

### Scanning Electron Microscopy (SEM) measurements

For obtaining SEM images of CD4+ T cells from heparinized normal donor whole blood, cryopreserved PBMC and lyophilized control cells, CD4+ lymphocytes were sorted using a FACSAria II sorter. These PBMCs were labeled with anti-CD3 APC, anti-CD4 FITC and anti-CD14 PB to collect only CD3 + CD4 + CD14- lymphocytes and to minimize the monocyte contribution. The sorted CD4+ T cells were then centrifuged at 350 g for 10 minutes and fixed overnight in 2% glutaraldehyde in PBS, pH 7.4. The CD4+ cells were further washed twice with PBS and resuspended in a small volume of PBS. A modified procedure from Singer [10] and Majstoravich [21] was used for coating CD4+ lymphocytes on glass slides. Briefly, a 20 μL of cell suspension was placed on a cover glass coated with 0.1% poly-L-lysine. After 30 minutes, cells on the cover glass were treated with the same glutaraldehyde fixative for 30 minutes. The cells were washed once with PBS and postfixed in 1% osmium tetroxide in PBS for 1 hour. After dehydration in ethanol, once with 70% for 10 minutes, once with 95% for 10 minutes and three times with 100% for 10 minutes, CD4+ cells on cover glass were treated by either critical point drying with liquid carbon dioxide or immersed in hexamethyldisilazane (HMDS; Aldrich, Atlanta, GA) for 5 min. The cover glasses treated with HMDS were placed in a vacuum desiccator at room temperature to be air-dried overnight.

The samples were imaged in an FEI Quanta 200 field emission environmental scanning electron microscope (FE-ESEM). All images were collected at 10 keV with a spot size of 2.5 μm. The specimen chamber was at full vacuum (<5 × 10^-3^ Pa), and the Everhart-Thornley secondary electron detector was used to form the images. Although some specimen charging did occur, the addition of 10 nm of gold to the surface (via plasma magnetron sputter coating) was sufficient to eliminate most charging effects. Beam damage was reduced by integrating the signal over eight short scans, rather than a single, longer scan. The image scale was calibrated according to the Geller Magnification Reference Standard. All images were saved in the 256 gray scale tagged image format (.tif).

### Comparison of SEM image characteristics

Based on the visual observations of key biological descriptors such as the density and shape of microvillis, we pursued the quantitative SEM image analyses by estimating cell surface roughness as an approximation to microvilli density/coverage, shape/geometry and length. Although SEM images do not provide direct 3D surface measurements, 3D depth maps can be derived by using photogrammetry or stereo (shape from shading) techniques [22] for their applications to SEM [23]. Unfortunately, these techniques cannot be applied to biological samples since they require multiple acquisitions while the sample may be altered or damaged after one image acquisition. Nevertheless, we assume that the SEM image shades of gray correlate with the thickness of microvillis at the tangential plane to a cell (e.g., with respect to a frontal view). We also assume that the cell surface at the root of microvillis does not change its composition (and hence SEM intensities) across the tangential plane to a cell. Under these assumptions, we can evaluate the cell surface roughness by computing a geodesic surface area in either horizontal or vertical direction. The area computation can be implemented by adding intensity increments along either rows or columns as shown in Eq. 3.
3

where A is the area, and I is the intensity of the SEM image. Row and Col entries are constrained to an image subarea representing the tangential plane to a cell. Finally, the statistics of all surface area measurements over each category (whole blood, PBMC, and Cyto-Trol) of images can be compared to determine the differences between whole blood and the other two cell categories.

## Electronic supplementary material

Additional file 1: **Sequence of human CD4 receptor protein and four peptide sequences within the extracellular domains of the CD4 receptor protein detected by MRM MS method.** (DOC 22 KB)

## Abbreviations

CD4: Cluster of differentiation 4
PBMC: Peripheral blood mononuclear cells
ABC: Antibody bound per cell
MRM MS: Multiple reaction monitoring mass spectrometry
SEM: Scanning electron microscopy
HMDS: Hexamethyldisilazane
PDF: Probability distribution function.
