# Supplementary material for: Quantifying CD4 receptor protein in two human CD4+ lymphocyte preparations for quantitative flow cytometry
Source: Clin Proteomics. 2014 Dec 11;11(1):43. doi: 10.1186/1559-0275-11-43 (PMC4277840; doi:10.1186/1559-0275-11-43)
Supplement: Supplementary file 1 — Additional file 1: Sequence of human CD4 receptor protein and four peptide sequences within the extracellular domains of the CD4 receptor protein detected by MRM MS method. (DOC 22 KB) [file 12014_2014_85_MOESM1_ESM.doc]

Appendix

Sequence of human CD4 receptor protein and peptide sequences detected by MRM MS method are shown below. Membrane associated and cytoplasmic peptides are italicized, extracellular domain D1 is colored in blue, and peptides detected by MRM MS in the four extracellular domains are in bold font (P1: ILGNQGSFLTK; P2: SLWDQGNFPLIIK; P3: ASSIVYK; P4: ATQLQK).

KKVVLGKKGDTVELTCTASQKKSIQFHWKNSNQIK**ILGNQGSFLTK**GPSKLNDRADSRR**SLWDQGNFPLIIK**NLKIEDSDTYICEVEDQKEEVQLLVFGLTANSDTHLLQGQSLTLTLESPPGSSPSVQCRSPRGKNIQGGKTLSVSQLELQDSGTWTCTVLQNQKKVEFKIDIVVLAFQK**ASSIVYK**KEGEQVEFSFPLAFTVEKLTGSGELWWQAERASSSKSWITFDLKNKEVSVKRVTQDPKLQMGKKLPLHLTLPQALPQYAGSGNLTLALEAKTGKLHQEVNLVVMR**ATQLQK**NLTCEVWGPTSPKLMLSLKLENKEAKVSKREKAVWVLNPEAGMWQCLLSDSGQVLLESNIKVLPTWSTPVQP*MALIVLGGVAGLLLFIGLGIFFCVRCRHRRRQAERMSQIKRLLSEKKTCQCPHRFQKTCSPI.*
